# *In silico* characterization and Molecular modeling of double-strand break repair protein *MRE11* from *Phoenix dactylifera* v deglet nour

**DOI:** 10.1186/s12976-015-0013-2

**Published:** 2015-11-05

**Authors:** Imen Rekik, Zayneb Chaabene, C. Douglas Grubb, Noureddine Drira, Foued Cheour, Amine Elleuch

**Affiliations:** Laboratory of plant biotechnology, Faculty of sciences of Sfax, University of Sfax, Sfax, Tunisia; Leibniz Institute of Plant Biochemistry, Halle, Germany; High Institute of Applied Biology of Medenine, Medenine, Tunisia

## Abstract

**Background:**

DNA double-strand breaks (DSBs) are highly cytotoxic and mutagenic. *MRE11* plays an essential role in repairing DNA by cleaving broken ends through its 3′ to 5′ exonuclease and single-stranded DNA endonuclease activities.

**Methods:**

The present study aimed to *in silico* characterization and molecular modeling of *MRE11* from *Phoenix dactylifera* L cv deglet nour (*DnMRE11*) by various bioinformatic approaches. To identify *DnMRE11* cDNA, assembled contigs from our cDNA libraries were analysed using the Blast2GO2.8 program.

**Results:**

The *DnMRE11* protein length was 726 amino acids. The results of HUMMER show that *DnMRE11* is formed by three domains: the N-terminal core domain containing the nuclease and capping domains, the C-terminal half containing the DNA binding and coiled coil region. The structure of *DnMRE11* is predicted using the Swiss-Model server, which contains the nuclease and capping domains. The obtained model was verified with the structure validation programs such as ProSA and QMEAN servers for reliability. Ligand binding studies using COACH indicated the interaction of *DnMRE11* protein with two Mn^2+^ ions and dAMP. The ConSurf server predicted that residues of the active site and Nbs binding site have high conservation scores between plant species.

**Conclusions:**

A model structure of *DnMRE11* was constructed and validated with various bioinformatics programs which suggested the predicted model to be satisfactory. Further validation studies were conducted by COACH analysis for active site ligand prediction, and revealed the presence of six ligands binding sites and two ligands (2 Mn^2+^ and dAMP).

**Electronic supplementary material:**

The online version of this article (doi:10.1186/s12976-015-0013-2) contains supplementary material, which is available to authorized users.

## Introduction

The palm family emerged ~80 million years ago and represents one of the lineages that radiated early in monocot evolution [1]. The genomic comparative analysis of date palm with other species presents an ideal opportunity to investigate the dynamics of angiosperm gene family evolution. The integrity of the genome is constantly threatened by environmental influences and cellular metabolic processes. DNA double strand breaks (DSBs) are among the most hazardous of all DNA lesions and arise from failures in genome metabolism processes and from exogenous sources. In addition they are important programmed intermediates in DNA metabolism.

DNA double-strand breaks are highly cytotoxic and mutagenic [2]. DSBs can arise during replication and as products of ionizing radiation and genotoxic chemicals, but are also endonucleolytically generated intermediates in meiosis, mating type switching, and recombination [3]. DSBs are predominantly repaired by two pathways. Nonhomologous end joining directly rejoins DSBs, whereas homologous recombination utilizes a sister chromatid or homologous chromosome as a template for DNA resynthesis and rejoining [4]. The *MRE11*-Rad50-Nbs1 (MRN) complex is a keystone complex that recognizes double-strand break (DSB) damages and responds with nonhomologous end joining (NHEJ) and homologous recombination (HR) pathways [5, 6]. In addition to the repair of DNA DSBs and cell cycle checkpoint signaling, the MRN complex plays an important role in telomere maintenance, mating type switching, meiotic recombination, and suppression of gross chromosomal rearrangement [7]. *MRE11* plays an essential role in repairing DNA by cleaving broken ends through its 3′ to 5′ exonuclease and single-stranded DNA endonuclease activities, as well as hairpin nuclease activities [8]. In addition, *MRE11* provides a surface for other DNA repair proteins and checkpoint factors which link the *MRE11* complex activities to a wide variety of cellular processes [9]. Structural studies of archaeal, bacterial and human *MRE11* homologs have revealed that *MRE11* forms a dimer. These *MRE11* homologs consist of the nuclease domain containing the active site and the capping domain, which provides selectivity concerning DNA substrates, and they dimerize through the interaction between the two helices by forming a four helix bundle [10, 11]. The dimerization of *MRE11* is crucial as it functions as a frame for Rad50 and DNA binding [10, 11]. Nbs1 (also known as Nibrin or p95) is only present in the eukaryotic *MRE11* complex. Nbs1 plays key roles in the DNA-damage checkpoint signaling functions of the MRN complex through interactions with a number of proteins, such as Mdc1 (mediator of the DNA-damage checkpoint 1) and ATM [12]. *MRE11* from eukaryotes is formed by two regions: the N-terminal core domain containing the nuclease and capping domains, and the C-terminal half containing the DNA binding and GAR domains [5, 6]. While the N-terminal domain, which is responsible for Nbs1 binding and nuclease activity, is conserved in all species, the C-terminal domain is distinct only in eukaryote *MRE11* [5, 6]. The *MRE11* gene has been identified in the genomes of all of the eukaryotes sequenced to date, including the Arabidopsis *MRE11* ortholog [13]. The homology between different *MRE11* orthologs is the strongest in the N terminus which contains four conserved phosphoesterase domains, but is less pronounced in the C terminus of the protein which contains two DNA binding domains [14]. The N-terminal region harbors an Nbs1 interacting domain [15], while at the C-terminal region interacts with Rad50 [8]. Originally, *MRE11* was identified in yeast (*S. cerevisiae*) as a gene required for early steps of meiotic recombination, namely for induction as well as for repair of meiotic DSBs [16]. In this study, we present and analyze for the first time an *in-silico* characterization and homology modelling of *MRE11* from *Phoenix dactylifera* v deglet nour (*DnMRE11*) by various bioinformatic approaches, including motif analysis, secondary structure prediction, 3D structure analysis and phylogenetic tree construction.

## Materials and methods

**Fig. 1:**
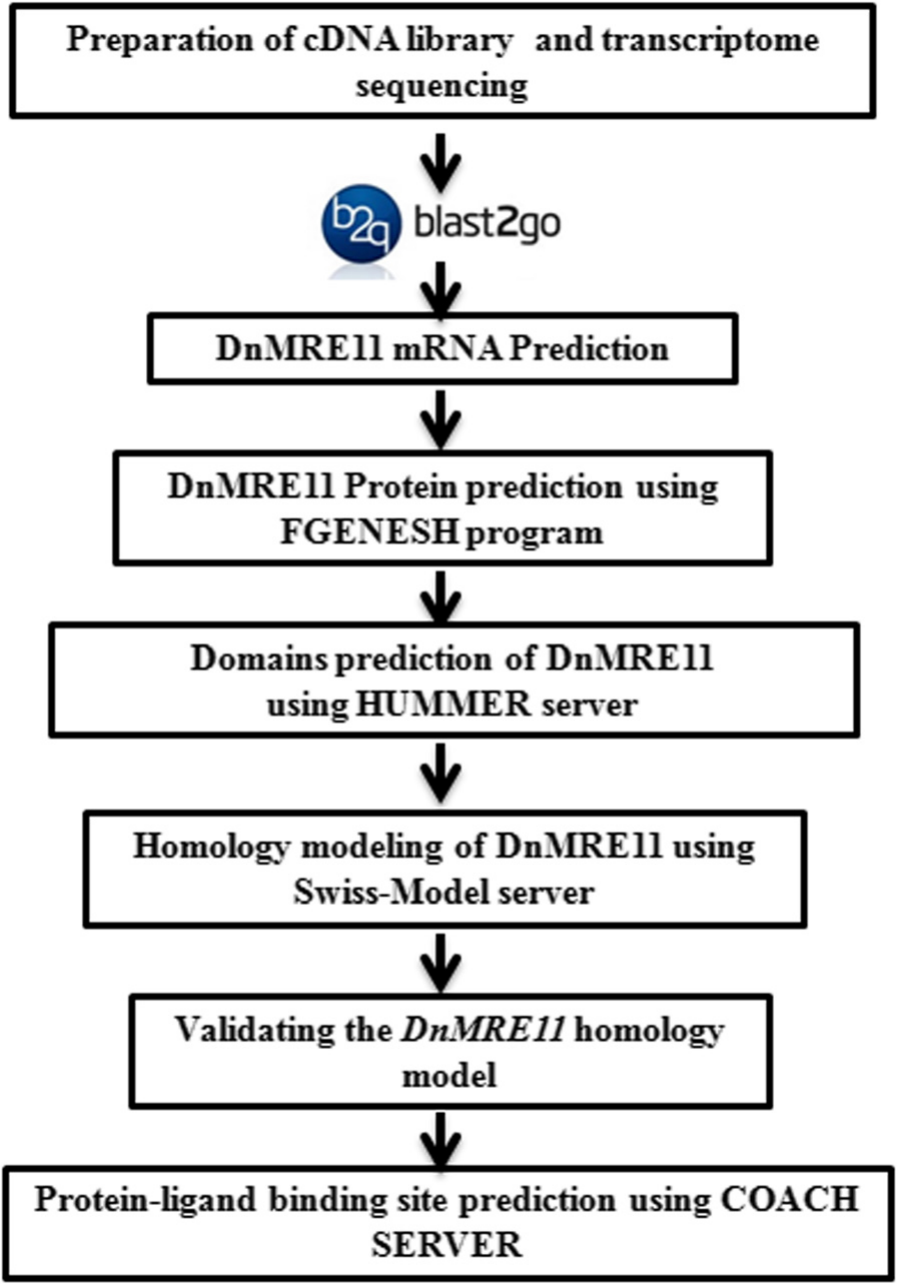
Overall protocol of DnMRE11 model prediction

For homology model prediction of DnMRE11, we have developed a procedure which combined old protocols employed in the previous works. Figure 1 show the overall protocol of DnMRE11 model prediction.

### RNA preparation

Total RNA was extracted by modified CTAB method [17] from young leaves of *Phoenix dactylifera* which were snap-frozen and stored at −70 °C until processing. RNA integrity was confirmed using the Agilent 2100 Bioanalyzer with a minimum integrity number value of 8. Equal amounts of total RNA from each tissue were pooled together for cDNA preparation.

### Preparation of cDNA library for transcriptome sequencing

The poly (A) RNA was isolated from 20 μg of the total RNA pool using Dynal oligo (dT) 25 beads (Invitrogen) according to the manufacturer’s protocol. Following purification, the mRNA was fragmented into smaller pieces at 70 °C for 5 min in the fragmentation buffer (Ambion) and reverse-transcribed to synthesize first strand cDNA using SuperScript III reverse transcriptase (Invitrogen) and N6 random hexamers (Takara). Subsequently, second strand cDNA was synthesized using RNase H (Invitrogen) and DNA polymerase (Invitrogen). These cDNA fragments were further processed by end repair using T4 DNA polymerase, the Klenow fragment of DNA polymerase, and T4 polynucleotide kinase (NEB), and ligation of adaptors with Illumina’s adaptor oligo mix and T4 DNA ligase (Invitrogen). The products were gel purified to obtain DNA approximately 200 bp long using Qiaquick Gel Extraction Kit (Qiagen) and enriched with PCR for preparing the sequencing library. The quality of the cDNA library was examined by Agilent 2100 Bioanalyzer.

### Illumina sequencing

The cDNA library was sequenced from both of 5′ and 3′ ends on the Illumina GA IIx platform according to the manufacturer’s instructions. The conversion of the fluorescent images to sequences, base-calling and quality value calculation were performed by the Illumina data processing pipeline (version 1.4), in which 75 bp paired-end reads were obtained. EST reads obtained from sequencing were cleaned using Seqclean program and assembled by CAP3 [18] using default settings. After assembly, to identified *DnMRE11* cDNA, a local BLASTX [19] was used to compare the assembled contigs and singletons against the NR database and analysed using Blast2GO2.8 [20] to provide Gene Ontology, BLAST and domain/Interpro annotation. Candidate mRNA for *DnMRE11* from *Phoenix dactifera* v degelt nour were identified *in silico* using FGENESH prediction (http://www.softberry.com; with the monocot matrix). Evaluation of *DnMRE11* predicted protein was done based on the identification of domains in the NCBI Conserved Domains Database (CDD), phytozome of June 2013 (http://www.phytozome.net/) and the most recent version of HMMER (HMMERV3.0; [21]).

### Sequence alignments

The *DnMRE11* protein sequence was submitted to profile-sequence searches with NCBI, phytozome of June 2013 and most recent version of HMMER. We recovered fifty *MRE11* proteins of plants. Protein alignments were performed using MUSCLE [22]. *MRE11* proteins were prefixed with the corresponding genus and species initials. Phylogenetic trees were constructed using Phyml software [23] based on the sequence of *MRE11* to determine the distribution and evolutionary trend of *MRE11* in plants using the Maximum likelihood method with 1000 bootstrapping replicates. The phylogram was generated using EvolView software [24]. After alignement, the fifty *MRE11* proteins of plants were submitted to the ConSurf server (http://consurf.tau.ac.il/) for analysis. The ConSurf server assigns relative conservation scores to each residue, taking into account the evolutionary relationships among the family of homologs. The scores are normalized such that the average score is zero, and negative and positive deviations represent the degrees of conservation and variation, respectively. Each residue is then assigned a value 1–9 (1 for most variable, 5 for average, up to 9 for most conserved), which is used for mapping the relative conservation on the molecular surface (see Figure legends). Sequence alignment of *DnMRE11*, *Aeropyrum pernix* K1 (Aep*MRE11*, archaea), *Homo sapiens* (Hms*MRE11*, animals), *Kocuria sp*. strain *UCDOTCP* (Koc*MRE11*, bacteria), *Saccharomyces cerevisiae* (Sac*MRE11*, fungi) and *Galdieria sulphuraria* (Gas*MRE11*, protista) was done by ClustalX and viewed with CLC Genomics Workbench (http://www.clcbio.com/).

### Homology modeling

PDB file of DnMRE11 protein was generated by Swiss-Model server (http://www.expasy.org/swissmod/SWISS-MODEL.html). In order to build a model of protein domain, Multiple Sequence Alignment was performed between full length DnMRE11 protein sequence and another protein domain sequence in this database. To build the model of the DnMRE11 protein with more homology, high resolution (1.80 A) structure of DnMRE11 protein model in Swiss model server was selected as template.

### Model reputation

The backbone conformation of the modeled structure of *DnMRE11* with ligand was calculated by analyzing the phi (Φ) and psi (ψ) torsion angles using Ramachandran plot v 2.0 (http://dicsoft1.physics.iisc.ernet.in/rp/select.html), as determined by Ramachandran plot statistics. The model was further analyzed by, QMEAN [25] and ProSA [26]. ProSA was used for the display of Z-score and energy plots. The volume area dihedral angle for fractional accessible surface area were done with VADAR (http://vadar.wishartlab.com/).

We used COACH [27] for protein–ligand-binding site prediction for structure-based biological function annotation of *DnMRE11*. Predicted models were further refined using a side-chain refinement protocol of Discovery Studio 3.5.

## Results and discussion

The *MRE11*/Rad50 (MR) complex plays a key role in DSB repair. Homologs of *MRE11* and Rad50 are found in all kingdoms of life and are essential for genome integrity [28]. After BLAST2GO analyse and searching in annotation results with the keyword “double-strand break repair protein” from the 24,071 transcripts, one *MRE11* cDNA was identified (contig 6335). This cDNA has a significant homology with the *MRE11* gene of *Phoenix dactylifera* v Khalas and other species with more than 90 % sequence similarity (Additional file 1). The results of BLASTX and annotation show that this cDNA is a potential candidate gene of the *DnMRE11*.

### Sequence analysis of DnMRE11 protein

**Fig. 2:**
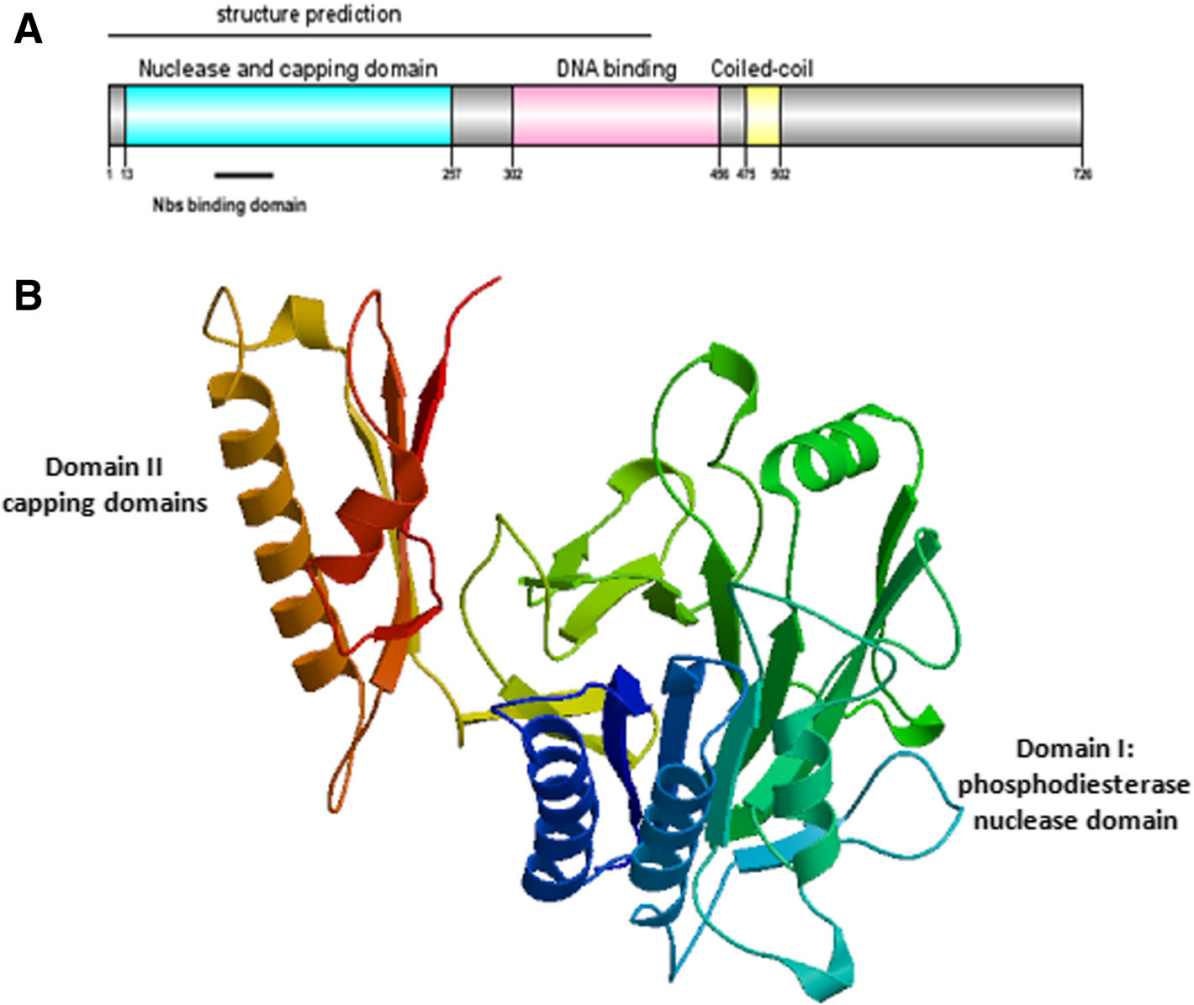
**a** Domain features of *DnMRE11*; (**b**) 3D structure of *DnMRE11*

The *DnMRE11* predicted protein length was 726 amino acids. The molecular mass was 81,54 kD, and isoelectric point of this protein was 6,25. The predicted localization for the Eukarya domain of *DnMRE11* by Predict Protein server was nucleus (GO term ID: GO:0005634). The results of NCBI CDD, pfam and HUMMER analyses show *DnMRE11* is formed by three domains: the N-terminal core domain containing the nuclease and capping domains (13 aa, 257 aa), the C-terminal half containing the DNA binding (302 aa, 456 aa) [5, 6] and a coiled coil region with a hydrophobic surface (Fig. 2a). This specific location on this coiled-coil region interacts with adjacent *MRE11* and DNA binding sites on Rad50 and suggests a mechanism for ATP-dependent control of the *MRE11* exonuclease by Rad50, by unwinding and/or repositioning DNA ends into the *MRE11* active site [5, 6]. While the N-terminal domain, which is responsible for Nbs1 binding and nuclease activity, is conserved in all species, the C-terminal (DNA binding and coil) domain is distinct only in eukaryotic *MRE11* [5, 6].

### Homology model structure analysis of DnMRE11 protein

The Swiss-Model server was used to predict the 3D sturcture of *DnMRE11* based on known crystal structures of homologous proteins (Fig. 2b). The lack of a 3D structure for *MRE11* in PDB motivated us to construct the 3D model for *MRE11*. The most successful techniques for prediction of three dimensional structures of proteins rely on aligning the sequence of a protein of to a homolog of known structure. The highest-scoring and validated model for *DnMRE11* that exhibits the greatest amino acid sequence identity with the crystal structure is double-strand break repair protein *MRE11* of *Schizosaccharomyces pombe* Scp*MRE11* (PDB ID : 4FBK1.A), which is in the *MRE11* superfamily. Only 404 residues of the N terminus (nuclease and capping domains) of *DnMRE11* have modelled with 100.0 % confidence by the single highest scoring template (Fig. 2b). This protein is 43.33 % identical to the DnMRE11 protein across 54 % of amino acid sequence. The alignement of two secondary structures of DnMRE11protein and the best template PDB : 4FBK1.A is shown in Additional file 2.

The stereochemical qualities of the predicted models of DnMRE11 proteins were analysed through QMEAN and ProSA servers confirmation was evaluated by the inspection of the Psi/Phi Ramachandran plots.

**Fig. 3:**
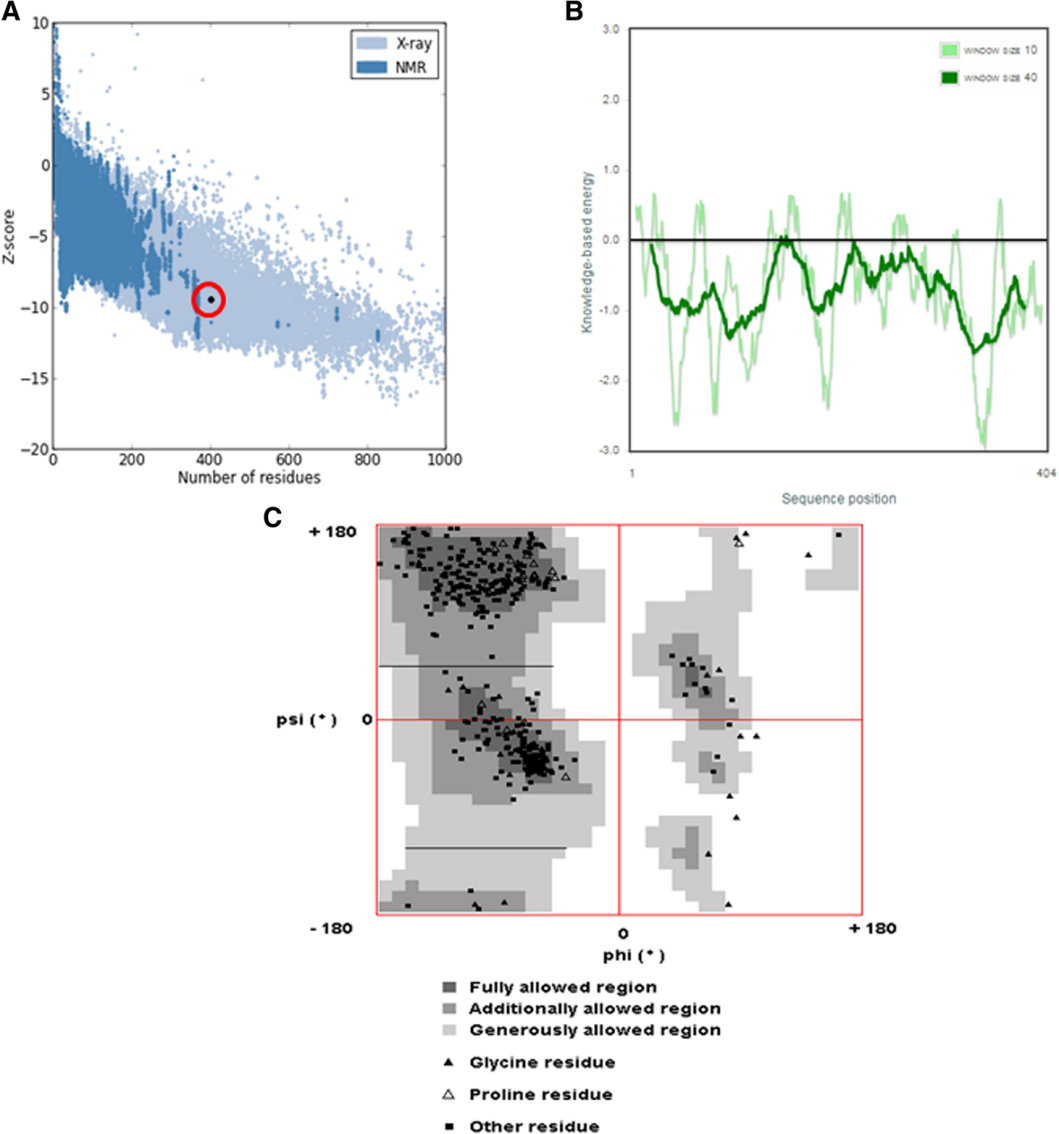
**a**, **b** and **c** Structural validation of the *DnMRE11* model using ProSA web tool. **a** ProSA overall Z score,9.5 is indicated in graph as black dot. A negative value of overall energy profile confirmed the reliable structural conformation of *DnMRE11*. **b** Energy profile of the *DnMRE11* homology model. **c** Ramachandran plot of the *DnMRE11* model showing 80 % of amino acid residues in the core region (*dark gray*)

ProSA was used to check the three- dimensional model of DnMRE11 proteins for potential errors. The program displays 2 characteristics of the input structure: its Z-score and a plot of its residue energies. The ProSA Z-score of −9.5 indicates the overall model quality of DnMRE11 protein (Fig. 3a). Z-score also measures the deviation of total energy of the structure with respect to an energy distribution derived from random conformations. The scores indicate a highly reliable structure and are well within the range of scores typically found for proteins of similar size. The energy plot shows the local model quality by plotting knowledge-based energies as a function of amino acid sequence position (Fig. 3b).

QMEAN analysis was also used to evaluate and validate the model.

The QMEAN4 score of the model was 0.672 and the Z-score was −1.57 which was close to the value of 0 and this shows the good quality of the model because the estimated reliability of the model was expected to be in between 0 and 1 (Table 1). A comparison between normalized QMEAN score (0.672) and protein size in non-redundant set of PDB structures in the plot revealed different set of Z-values for different parameters such as C-beta interactions (0.38), interactions between all atoms (−0.23), solvation (−0.49) and torsion (−1.45) (Table 1).

**Table 1:** Z scores and energy of individual component of QMEAN for DnMRE11 model

Scoring function term	Energy	Z-score
C-beta interactions	−170.63	0.38
All-atom pairwise	−10397.83	−0.23
Solvation	−32.72	−0.49
Torsion angle	−76.09	−1.45
QMEAN4 score = 0.672		−1.57

The constructed homology model was also evaluated for structural and stereo chemical efficiency. A Ramachandran phi-psi plot for *DnMRE11* (Fig. 3c) revealed that 80 % of residues lay in the core region (dark gray), another 15,5 % were in the allowed region (light gray), 2,5 % were in generally region (very light gray) and only 2 % lay in the disallowed region (white). The above analysis of the predicted structure provides solid evidence that the predicted 3D structure of *DnMRE11* is of good quality.

The overall structure of *MRE11* proteins of fungi (*Schizosaccharomyces pombe*) Scp*MRE11* (PDB ID:4fbk) [29] and human Hm*MRE11* (PDB ID: 3t1i) [30], are relatively similar compared with *DnMRE11*. Both nuclease and capping domain structures are present and the structure of the *DnMRE11* nuclease domain is more similar to the equivalent domain from these *MRE11* proteins (Fig. 2b) [10, 11].

The *DnMRE11* core comprises two α/β fold domains, a larger N-terminal nuclease domain and a smaller C-terminal capping domain (Fig. 2b, Additionnal file 2). The *DnMRE11* nuclease domain, which resembles the calcineurinlike Ser/Thr phosphosesterase, consists of five helices and 13 strands, and the capping domain is composed of three strands packed by two helices on one face (Additionnal file 2).

Domain II, which consists of a three-stranded β sheet and two α helices, partially caps the active site phosphodiesterase motifs of Domain I, suggesting that Domain II plays a role in DNA substrate specificity (Fig. 2b). This Domain II cap appears to be a unique *MRE11* feature as no equivalent domain or fold is found in the protein phosphatases. In the capping domain of *DnMRE11*, substantial differences exist in the length and orientations of the loops compared with those of template Scp*MRE11*. In general, helices and loops in the *DnMRE11* capping domain are relatively longer than those of Scp*MRE11* (Additionnal file 2, β 15).

These results show that the *DnMRE11* capping domain with three strands is closer to the canonical structure [10, 11].

### Active binding site prediction of DnMRE11

A multiple-sequence alignment revealed that the *DnMRE11* protein has low similarity with homologs from others species, such as *Aeropyrum pernix* K1 (Aep*MRE11*, archaea), *Homo sapiens* (Hms*MRE11*, animals), *Kocuria sp*. strain *UCD*-*OTCP* (Koc*MRE11*, bacteria), *Saccharomyces cerevisiae* (Sac*MRE11*, fungi) and *Galdieria sulphuraria* (Gas*MRE11*, protista), with the exception of active site residues (Additional file 3). Domain I contains five conserved phosphodiesterase motifs, which form the nuclease active site [30] (Additional file 1). Predection of the active site location of *DnMRE11* protein by the DEPTH server (Additional file 4) showed the phosphodiesterase motifs (red color) situated between the nuclease and capping domain. The Domain I fold and active site location resemblethe catalytic domain of calcineurin-like Ser/Thr phosphatases and the DNA base excision repair enzyme apurinic endonuclease 1 (APE1). This resemblance suggests that the di-metal nuclease mechanism of *MRE11* is similar to the di-metal protein phosphatase mechanism of Ser/Thr phosphatases [31].

The active site of any protein is critical for its activity, thus blocking it with a suitable ligand may result in inhibition of the protein either partially or completely. In this regard, it becomes highly essential to determine the amino acid residues of the protein that forms the active site.

**Fig. 4:**
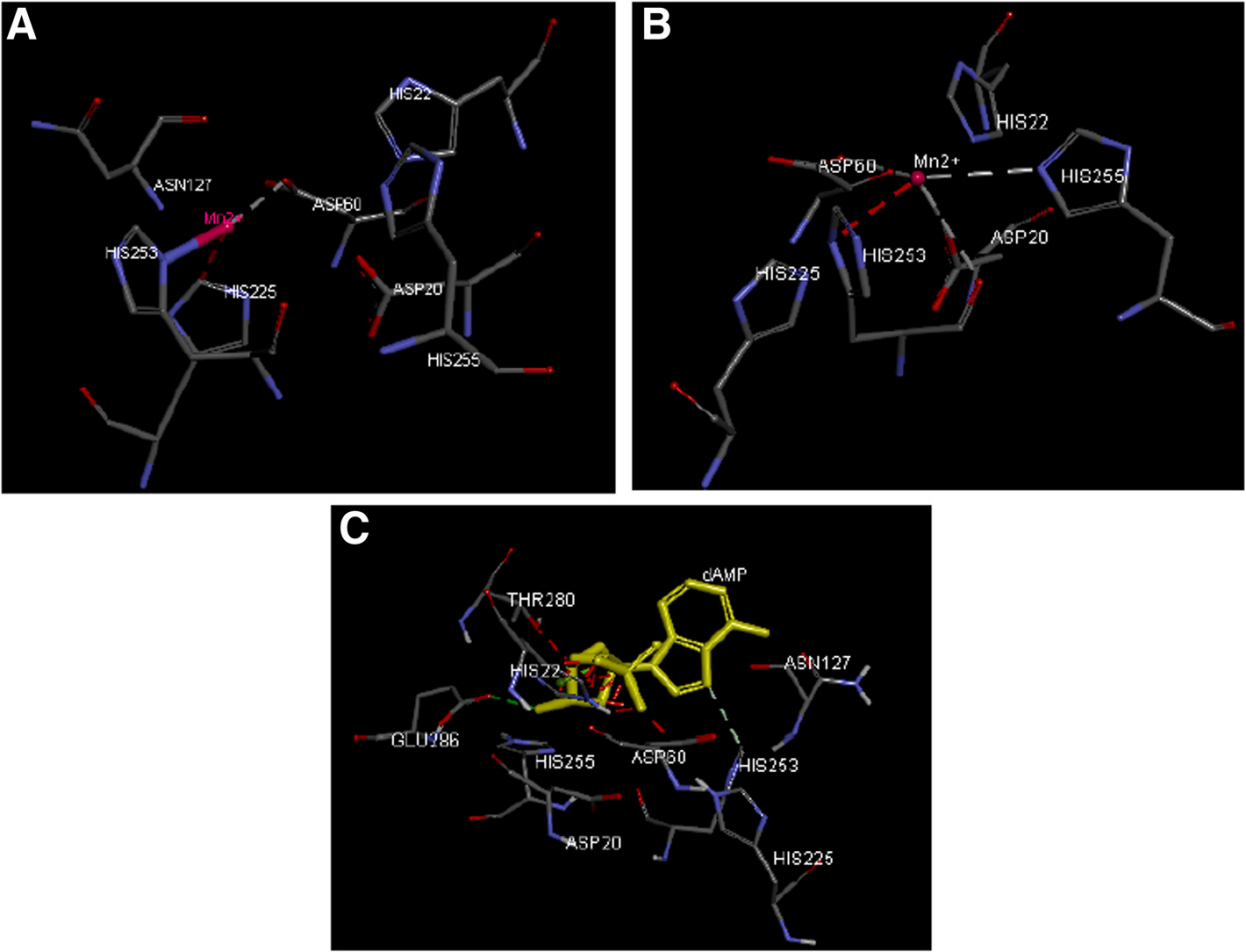
The 3D structure of *DnMRE11* showing its binding site. The proposed binding modes of Mn2+ and dAMP molecules are shown in stick format and noncarbon atoms are colored by atom type. Critical residues for binding of the first Mn2+ (**a**), second Mn2+ (**b**) and dAMP (**c**) are shown in this figure. The pink balls corresponding to the Mn 2+ ions and dAMP molecule are colored in yellow. Hydrogen bonds are shown in this figure with dotted lines

The COACH server analysis demonstrated that two Mn2+ ions are coordinated in each of the two apparently functional nuclease sites and the crucial amino acid residues forming the active site of *DnMRE11* include ASP20, ASP60, ASN127, HIS225, HIS253 for the one Mn2+ ion (Fig. 4a) with derict interraction with residues ASP60, HIS225 and HIS253 (Fig. 4a). The second Mn2+ interract with ASP20, ASP60, HIS253 and HIS255 (Fig. 4b). The COACH predicts also that the *MRE11* active site binds dAMP mainly via the phosphate moiety, which is bound by HIS22, ASP60, GLU286, HIS253, HIS255 and THR280 (Fig. 4c). The double coordination of the dAMP phosphate by both active site metals resembles the binding of phosphorylated protein residues in Ser/Thr phosphatases, further supporting a common phosphoesterase mechanism between *MRE11* and Ser/Thr phosphatases [31]. One conserved residue in eukaryotic *MRE11* proteins, Glu286 (Fig. 4c), forms H-bonds with HIS253 and stabilizes this histidine. The same active site binding substrates were found using the FunFOLD server [31] and the 3DLigandSite server [32].

Comparing structures of Hms*MRE11* (*Homo sapiens,* PDB : 3T1I) [30], Pf*MRE11* (*Pyrococcus furiosus*, archaea PDB ID: 1II7, [33]), Sp *MRE11* (*Schizosaccharomyces pombe* [29], PDB : 4fbkA) and *DnMRE11* protein, we conclude that the residues which bind the metals and dAMP are the same at the active site for all these species. While there are overall similarities between the active site of *DnMRE11*, bacterial and archaeal *MRE11* proteins, some differences are observed. These differences are largely limited to residues that interact with metal coordinating residues, and all these residues are conserved in eukaryotes.

When we compared with the Hms*MRE11*sequence, we found the residues Arg80, Asp86, Asn116, and Pro120 of *DnMRE11* that contribute to Nbs1 binding [29] are also conserved (Additional file 3), thus these residues may be involved in binding Nbs 1 by *DnMRE11*. The accessible surface areas in the amino acid sequence of *DnMRE11* were predicted using the VADAR servers for model prediction (Additional file 5). We have predicted the ASA of each amino acid in the sequence along with the fractional residual volume available for the amino acids in the main as well as in the side chains. The quality of the model with respect to the stereo packing and 3D profile quality were also predicted using the VADAR server. The area accessible to water molecules on the protein structure is said to be accessible surface area (ASA), which was measured in square angstroms or as fractional ASA ranging from 0 to 1.

Hydrophilic residues occupied a large fraction of ASA with hydrophobic residues forming only a small fraction (Additional file 5). ASA values both for the whole structure and side chains were predicted using VADAR. The majority of residues in *DnMRE11* have ASA scores less than 0.8, indicating tight folding the generally leaves residues inaccessible to water molecules. We estimated the accessible surface areas of all residues of the active site and the Nbs1 binding site that are involved in the *DnMRE11* 3D structure using the VADAR server.

The ASA scores of the active site (Mn 2+ binding sites) were near zero (Asp 20 : 0,01; Asp60 : 0.03; His225: 0; His253 : 0.22; His255 :0,17) indicating the residues are not accessible to water molecules and confirmed that domain II (the capping domain) hides the active site. However, Nbs binding sites are exposed to surface (ASA scores > 0,8).

### The plant MRE11 gene family

We selected 50 genes in 41 taxa that appear to belong to the *MRE11* family. The distribution of *MRE11* genes among the various species is shown in Table 2.

**Table 2:** List of accession numbers and list of taxa of plants used in this study

Viridiplantae		Ncbi accession code	Uniprot accession code
Chlorophyta			
Tetraselmis sp. GSL018	TspMRE11	JAC79210.1	
Micromonas sp. RCC299	MicMRE11		C1ECB4
Embryophyta			
Physcomitrella patens	PhpMRE11		G4XIR1
Magnoliophyta			
Amborella trichopoda	AmtMRE11		W1P0X4
Stem eudicotyledons			
Nelumbo nucifera	NenMRE11X1 NenMRE11X2	XP_010277930.1, XP_010277931.1	
Eudicotyledons			
Genlisea aurea	GeaMRE11		S8C042
Nicotiana tomentosiformis	NitMRE11X2	XP_009613836.1	
Nicotiana sylvestris	NisMRE11X2	XP_009787661.1	
Solanum tuberosum	SotMRE11like	XP_006341147.1	
Solanum lycopersicum	SolMRE11	XP_004246548.2	
Vitis vinifera	VivMRE11	XP_002281726.1	
Morus notabilis	MonMRE11	EXB89636.1	
Prunus mume	PrmMRE11	XP_008226714.1	
Malus domestica	MadMRE11X2	XP_008366397.1	
Fragaria vesca subsp. vesca	FrvMRE11like	XP_004294486.1	
Populus trichocarpa	PotMRE11	XP_006370340.1	
Ricinus communis	RicMRE11		B9SIE0
Cucumis sativus	CusMRE11like	XP_004154884.1	
Cucumis melo	CumMRE11X1, CumMRE11X2	XP_008454628.1 XP_008454629.1	
Medicago truncatula	MetMRE11	KEH38818.1	
Cicer arietinum	CiaMRE11likeX2, CiaMRE11X1	XP_004487655.1 XP_004487654.1	
Glycine max	GlmMRE11like	XP_003539581.1	
Eucalyptus grandis	EugMRE11	XP_010060498.1	
Citrus clementina	CicMRE11		V4URY3
Citrus sinensis	CisMRE11like	XP_006464669.1	
Eutrema salsugineum	EusMRE11		V4LH60
Brassica rapa	BrrMRE11	XP_009119960.1	
Capsella rubella	CarMRE11		R0EVY8
Arabidopsis thaliana	ArtMRE11	AED96476.1	
Liliopsida			
Musa acuminata subsp. Malaccensis	MuaMRE11X1 MuaMRE11X2	XP_009412711.1 XP_009412712.1	
Phoenix dactylifera v Deglet nour	DnMRE11		
Phoenix dactylifera v Khalas	KhMRE11	XP_008803852.1	
Zea mays	ZemMRE11	NP_001151499.1	
Sorghum bicolor	SobMRE11		C5YGR2
Setaria italica	SeiMRE11X1, SeiMRE11like	XP_004976962.1 XP_004974387.1	
Oryza brachyantha	OrbMRE11like	XP_006652883.1	
Oryza sativa Group Japonica	OrsMRE11		Q7XQR9
Brachypodium Distachyon	BrdMRE11like, BadMRE11	XP_003571409.1 XP_010240488.1	
Aegilops tauschii	AetMRE11	EMT05178.1	
Triticum turgidum	TrtMRE11		Q4GX62
Triticum aestivum	TraMRE11		W5B7S5
Hordeum vulgare	HovMRE11		F2DMY1

Phylogenetic analysis was performed on the multiply aligned plant *MRE11* proteins sequences, by the protein-maximum likelihood, using the *Saccharomyces cerevisiae MRE11* Sac*MRE11* out as an outgroup (Additional file 6). This analysis revealed the presence of two major clusters corresponding to separate *MRE11* gene subfamilies of monocots and eudicots species. Cluster 1 (blue) contains *MRE11* sequences identified in the Liliopsida (monocots) group. Cluster 2 (red) contains *MRE11* proteins from eudicots. A subcluster contained *MRE11* sequences that were found in stem eudicotyledons (grey). We found that Tsp*MRE11* and Mic*MRE11* (Chlorophyta, light blue), Php*MRE11* (Embryophyte, Green) and Amt*MRE11* (Magnoliophyta, yellow) are separated from monocot and eudicot proteins. We found many duplication events in this tree. In cluster I, two duplication events were found between the two paralogous *MRE11* proteins of the species *Phoenix dactylifera DnMRE11* (Deglet nour variety), Kh*MRE11* (Khalas variety) and between the *MRE11* paralogs of the species *Musa acuminata* (Mua*MRE11*X1, Mua*MRE11*X2). Two sequences, Sei*MRE11*X1and Sei*MRE11*- like, were identified which cluste red closely together within cluster 1, suggesting that these paralogues were generated by a lineage specific duplication event. Other duplication events were unresolved as in cluster II, *Cicer arietinum* (Cum*MRE11*X1, Cum*MRE11*X2) and *Cucumis melo* (Cum*MRE11*X1, Cum*MRE11*X2). Relationships among *MRE11* genes from monocots, stem eudicots, core eudicots, Chlorophyta, Embryophyte and Magnoliophyta were inferred from the conserved nuclease, capping and DNA binding regions. The ConSurf server was used to extract information about important residues, which are of functional value. This server provides evolutionary conservation scores for residues, which could be correlated with biological function. In our case, the ConSurf server predicted that residues of the active site and Nbs binding site have high conservation scores (dark pink in Table 3). Among these, D20 and P120 were found to have high scores, indicating evolutionary conservation and hence important functional roles (Additional file 7 and Table 3). Approximately 85 % of the residues are conserved among the nuclease and capping domains shown in Additional files 3 and 7.

**Table 3:** The table details the residue variety in % for each position in the query sequence. Each column shows the % for that amino-acid, found in position (‘pos’) in the MSA

Amino acid position of actif site	Pourcentage of concervation	Consurf grade
D20	100	9
H22	98	9
N127	98	9
H225	98	9
H253	98	9
H255	98	9
Amino acid position of Nbs binding site		
R80	94	9
D86	98	9
N116	98	9
P120	100	9

Most of the variation within this region occurs in the capping domain.

## Conclusion

*In silico* analysis of *DnMRE11* was conducted by motif analysis and phylogenetic tree construction using PhyML. The ConSurf server predicted that residues of the active site and Nbs binding site have high conservation scores. A model structure of *DnMRE11* was constructed using Swiss-Model server using homology-based modelling and validated with ProSA, QMEAN servers and Ramachandran plot analysis, which suggested the predicted model to be satisfactory. Further validation studies were conducted by COACH analysis for active site ligand prediction, and revealed the presence of six ligands binding sites and two ligands (2 Mn2+ and dAMP).

## Additional files

Additional file 1:
**Results of BLASTX and annotation of a potential candidate cDNA of the** ***DnMRE11.*** (PNG 148 kb) Additional file 2:
**Structure guided sequence alignment of** ***DnMRE11*** **with** ***Schizosaccharomyces pombe*** **(4fbk.1.A)** ***MRE11.*** (PNG 103 kb) Additional file 3:
**The alignment between** ***DnMRE11,*** ***Aeropyrum pernix*** **K1 (Aep** ***MRE11,*** **archaea),** ***Homo sapiens*** **(Hms** ***MRE11,*** **animals),** ***Kocuria*** **sp. UCD OTCP (Koc** ***MRE11,*** **bacteria),** ***Saccharomyces cerevisiae*** **(Sac** ***MRE11,*** **fungi) and** ***Galdieria sulphuraria*** **(Gas** ***MRE11,*** **protista).** Conserved residues are red. The five conserved phosphodiesterase motifs, which form the nuclease active site, are showns in this figure with roman numbers. (PNG 770 kb) Additional file 4:
**Prediction of the active site location of** ***DnMRE11*** **by the DEPTH server.** (PNG 133 kb) Additional file 5:
**Fractional accessible surface area of** ***DnMRE11.*** (PNG 94 kb) Additional file 6:
**Phylogenetic maximum likelihood tree showing the evolutionary relationships among** ***MRE11*** **proteins from 50 plant species.** Tree was created using the MUSCLE alignment tool and EvolView software. Bootstrap values are indicated in tree adjacent to the relevant branches. Eudicotyledons are highlighted in pink, Chlorophyta in light blue, Liliopsida (monocots) in blue, Embryophyta in green, Magnoliophyta in yellow, and stem eudicotyledons in grey. (PNG 186 kb) Additional file 7:
**Results of ConSurf analysis mapped onto the** ***MRE11*** **structure (residues 1–404) using the maximum likelihood method.** Shown on the *left* is the space filling view of the protein; *right*, the opposite side following a 180° rotation about the *y* axis. Conserved residues are darkest pink, variable residues are cyan, and others are white. (PNG 241 kb)
