# Supplementary figures and images for: In silico characterization and Molecular modeling of double-strand break repair protein MRE11 from Phoenix dactylifera v deglet nour
Source: Theor Biol Med Model. 2015 Nov 5;12:23. doi: 10.1186/s12976-015-0013-2 (PMC4635681; doi:10.1186/s12976-015-0013-2)

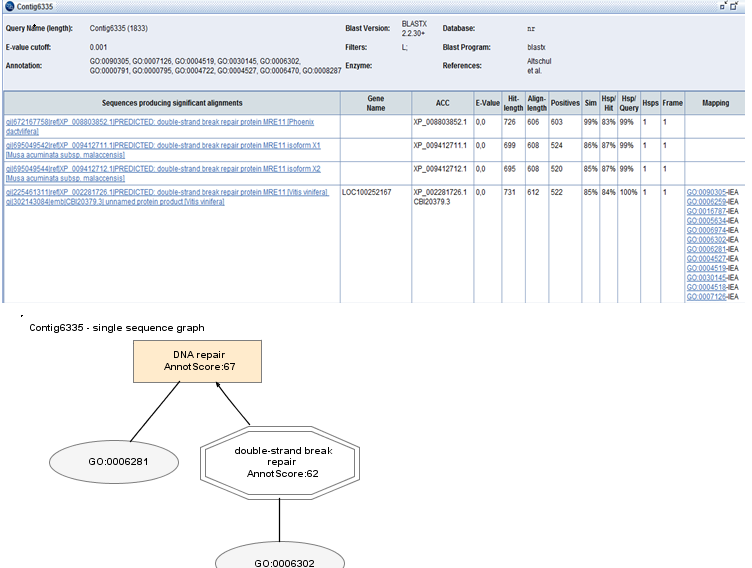

Supplement: Additional file 1: — Results of BLASTX and annotation of a potential candidate cDNA of the DnMRE11. (PNG 148 kb) [file 12976_2015_13_MOESM1_ESM.png]

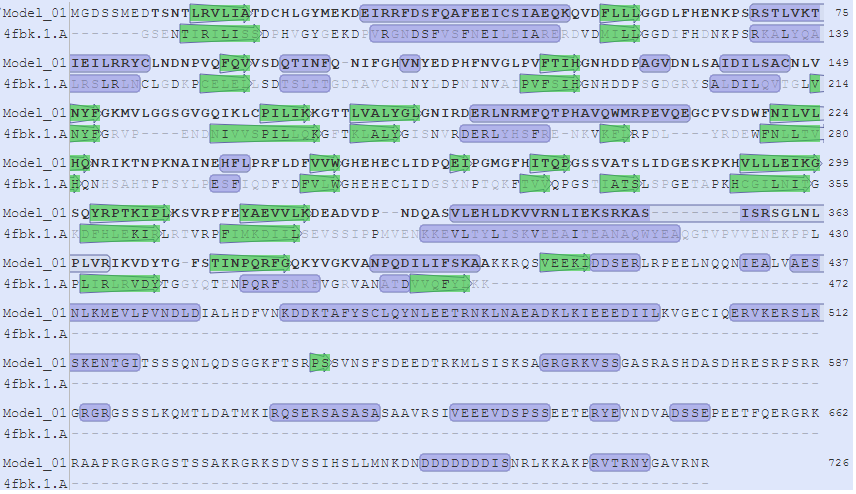

Supplement: Additional file 2: — Structure guided sequence alignment of DnMRE11 with Schizosaccharomyces pombe (4fbk.1.A) MRE11. (PNG 103 kb) [file 12976_2015_13_MOESM2_ESM.png]

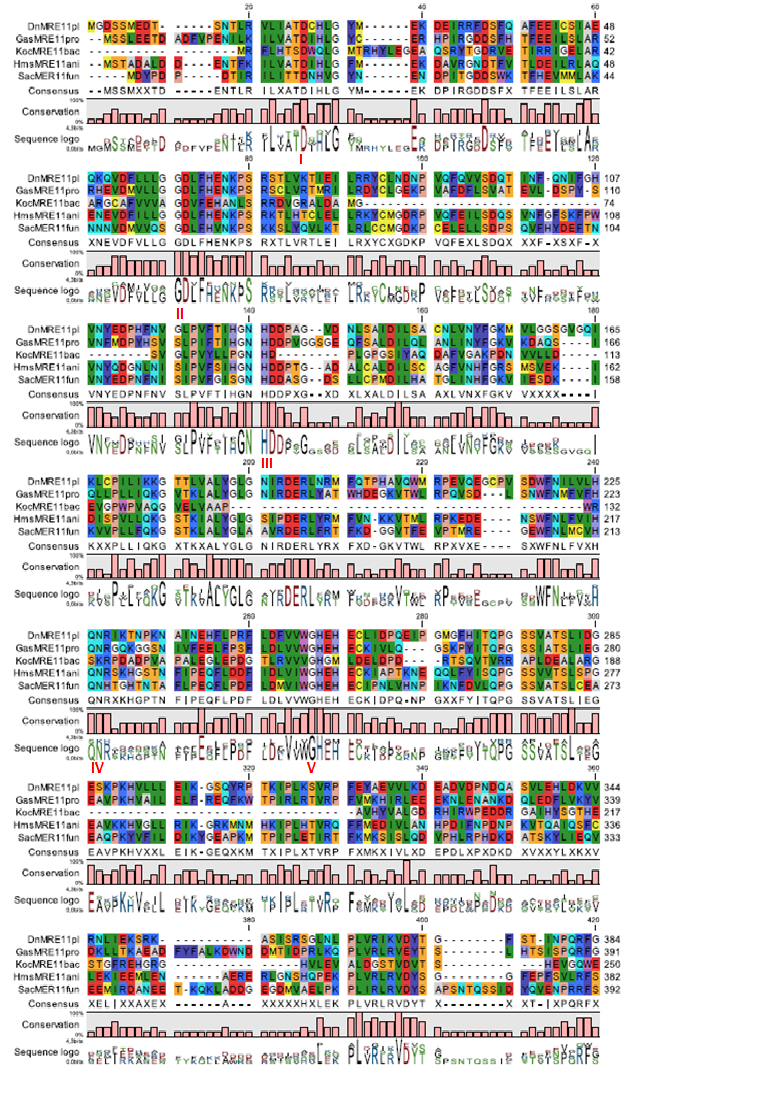

Supplement: Additional file 3: — The alignment between DnMRE11, Aeropyrum pernix K1 (Aep MRE11, archaea), Homo sapiens (Hms MRE11, animals), Kocuria sp. UCD OTCP (Koc MRE11, bacteria), Saccharomyces cerevisiae (Sac MRE11, fungi) and Galdieria sulphuraria (Gas MRE11, protista). Conserved residues are red. The five conserved phosphodiesterase motifs, which form the nuclease active site, are showns in this figure with roman numbers. (PNG 770 kb) [file 12976_2015_13_MOESM3_ESM.png]

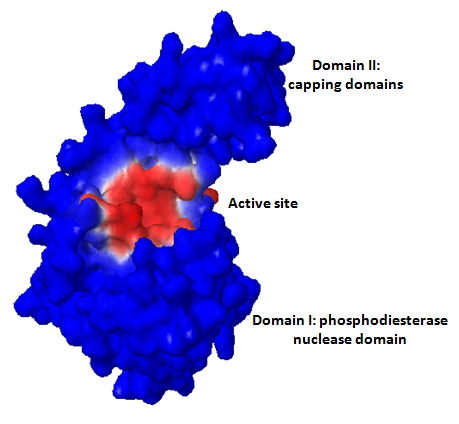

Supplement: Additional file 4: — Prediction of the active site location of DnMRE11 by the DEPTH server. (PNG 133 kb) [file 12976_2015_13_MOESM4_ESM.png]

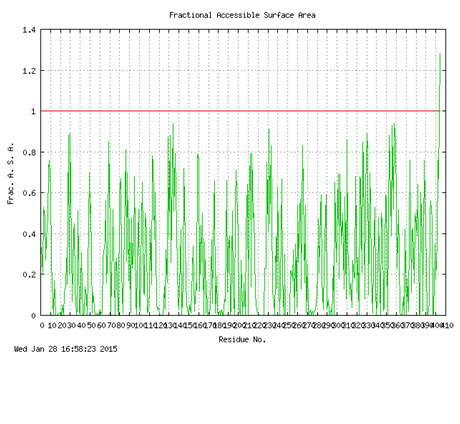

Supplement: Additional file 5: — Fractional accessible surface area of DnMRE11. (PNG 94 kb) [file 12976_2015_13_MOESM5_ESM.png]

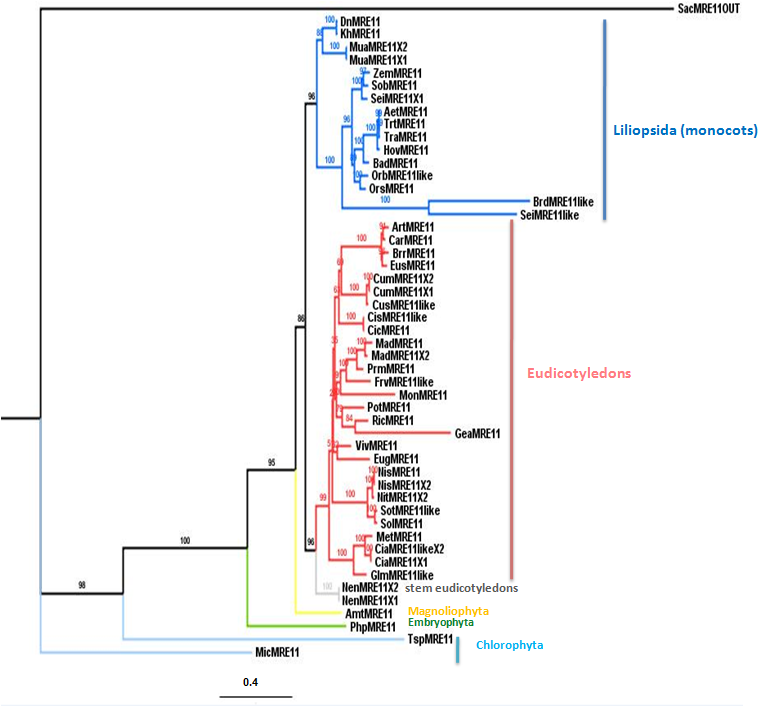

Supplement: Additional file 6: — Phylogenetic maximum likelihood tree showing the evolutionary relationships among MRE11 proteins from 50 plant species. Tree was created using the MUSCLE alignment tool and EvolView software. Bootstrap values are indicated in tree adjacent to the relevant branches. Eudicotyledons are highlighted in pink, Chlorophyta in light blue, Liliopsida (monocots) in blue, Embryophyta in green, Magnoliophyta in yellow, and stem eudicotyledons in grey. (PNG 186 kb) [file 12976_2015_13_MOESM6_ESM.png]

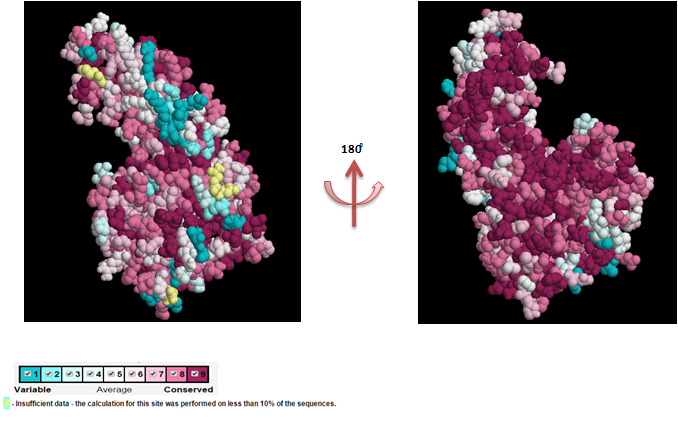

Supplement: Additional file 7: — Results of ConSurf analysis mapped onto the MRE11 structure (residues 1–404) using the maximum likelihood method. Shown on the left is the space filling view of the protein; right, the opposite side following a 180° rotation about the y axis. Conserved residues are darkest pink, variable residues are cyan, and others are white. (PNG 241 kb) [file 12976_2015_13_MOESM7_ESM.png]
